# The Utility of Acid-Fast Bacillus (AFB) and Fungal Cultures in Orthopaedic Infections

**DOI:** 10.7759/cureus.26639

**Published:** 2022-07-07

**Authors:** Jared Sanderford, Jacqueline Krumrey, Erin Campaigniac, Jason Lin, Peter Tsai, Olivia Pipitone

**Affiliations:** 1 Orthopaedic Surgery, Good Samaritan Regional Medical Center, Corvallis, USA; 2 Orthopaedics and Traumatology, Good Samaritan Regional Medical Center, Corvallis, USA; 3 Orthopaedic Surgery - Foot and Ankle, Good Samaritan Regional Medical Center, Corvallis, USA; 4 Orthopaedic Surgery - Hand and Upper Extremity, Good Samaritan Regional Medical Center, Corvallis, USA; 5 Graduate Medical Education - Biostatistics, Good Samaritan Regional Medical Center, Corvallis, USA

**Keywords:** orthopaedic infection risk factors, hand and foot infections, acid fast bacillus cutlures, fungal osteomyelitis, atypical cultures in orthopaedics, orthopaedic infection, orthopaedic afb cultures, orthopaedic fungal cultures

## Abstract

Introduction

When diagnosing suspected orthopaedic-related infections, fungal and acid-fast bacilli (AFB) cultures are often obtained intraoperatively. These cultures are difficult and time-consuming to grow and increase healthcare costs. This study aimed to quantify the rate of positive AFB and fungal cultures in orthopaedic infections and to compare potential risk factors for a positive result.

Methods

Orthopaedic surgical cases for suspected infection at one institution from March 2013 through December 2019 were included. Data were collected on patient demographics and procedure characteristics for patients with surgical AFB or fungal lab tests ordered on the day of surgery.

Results

Of the 813 patients for whom intraoperative AFB or fungal cultures were ordered, 3.8% (N=31) had a positive result. Of the 31 positive results, 30 were from fungal cultures and one was from AFB cultures. Patients with a positive versus negative culture result did not differ significantly by age, sex, American Society of Anesthesiologists (ASA) score, diabetes, obesity, or HIV/AIDS. In both unadjusted and adjusted analyses, peripheral vascular disease (PVD) was associated with higher odds of a positive fungal culture result (adjusted OR (aOR)=3.5, 95%CI=1.3-8.4). Likewise, in both unadjusted and adjusted models, a hand/foot operating region was associated with higher odds of a positive fungal culture result compared with all other regions (aOR=4.2, 95%CI=1.9-9.8).

Conclusion

Intraoperative fungal and AFB cultures may not need to be obtained except in orthopaedic surgical cases for hand or foot infections or in patients with PVD.

## Introduction

Infections are an unfortunate but inevitable reality of orthopaedic surgery. To focus on treatment for these infections, intraoperative cultures are obtained. One common approach to any orthopaedic-related infection is to perform a full panel of cultures including anaerobic/aerobic, fungal, and acid-fast bacillus (AFB) cultures. However, fungal and AFB cultures are rarely, if ever, positive [1,2]. For example, in aseptic prosthetic joint revisions, the rate of positive AFB and fungal cultures is 0% and 0.1% respectively [2]. Not only are they uncommon but they are also difficult to grow and require prolonged incubation, increasing healthcare costs [1].

There are very few studies that guide our usage of fungal and AFB cultures for orthopaedic infections. The literature for prosthetic joint infections (PJI) has provided us with the most studies on this topic, and even that is sparse. Kuruppu et al. report that PJI rates are between 0.86% and 1.03% at one and five years respectively [3]. Tokarski et al. evaluated the routine use of atypical cultures in the setting of presumed aseptic prosthetic joint revision and noted a low rate of true positive AFB and fungal cultures (0% and 0.1% respectively) [2]. Tokarski concluded that routine use of these cultures may not be warranted [2]. Bariteau et al. report the guidelines for routine testing for AFB and fungus in cases of aseptic revisions are lacking [1]. A retrospective review by Wadey et al. demonstrated a positivity rate for AFB and fungal cultures as 0% and 0.6% respectively after evaluating over 900 orthopaedic samples. Even in the microbiology literature, there are recommendations against obtaining AFB and fungal cultures, particularly in prosthetic joint infections due to the low positive rate [4].

Previous studies have evaluated risk factors for positive fungal culture results. These include substance abuse, presence of an indwelling catheter, parenteral hyperalimentation, diabetes mellitus, use of broad-spectrum antibiotics, HIV infection, and organ transplantation [1]. There have been several studies specifically looking at fungal infections in prosthetic joints. These studies have found similar risk factors for positive results. Azzam and Parvizi found several risk factors for positive culture results in prosthetic joints including prolonged antibiotics, chronic steroids, renal disease, malignancy, rheumatoid arthritis, diabetes, hepatic disease, and cardiac disease [5]. In evaluating comorbidities of patients with candidemia, Kao et al. found at least one of the following present in 90% of patients: chronic lung disease, diabetes, chronic heart conditions, stroke, history of alcoholism, liver disease, cancer, surgery within two months of diagnosis, organ transplant, rheumatologic disease, steroid use, HIV/AIDS, and prematurity [6]. Although this evaluated bloodstream infections, it provides guidance on possible risk factors for positive orthopaedic fungal infections.

Not only are these tests rarely positive, but they increase healthcare costs. Wadey et al. specifically looked at the economic impact of these laboratory tests [7]. They found that routinely obtaining five separate samples for each infection and sending them each for AFB, fungal, and aerobic/anaerobic cultures cost over $200,000 per year. The AFB and fungal cultures alone cost over $90,000 in one year.

The goal of the present study was to determine the utility of AFB and fungal cultures by evaluating the rate of positive AFB and fungal cultures in all operatively treated orthopaedic infections at one institution. Demographics and risk factors of patients with a positive AFB/fungal culture were compared to those with a negative AFB/fungal culture to identify characteristics that may increase the risk of a positive result. We hypothesized that the rate of positive AFB and fungal cultures for orthopaedic infections in this study would be less than 1% based on previous research. We also hypothesized that certain medical comorbidities such as diabetes, peripheral vascular disease, and HIV would increase the odds of obtaining a positive AFB/fungal culture result based on the studies previously discussed.

## Materials and methods

This study was approved as "exempt" by the Samaritan Health Services Regional Institutional Review Board as study data were retrospectively collected from electronic medical records (EMR). All completed orthopaedic surgical cases between March 2013 and December 2019 with an AFB and/or fungal culture ordered on the same day of the surgery were included. Orthopaedic cases were defined by the surgeon and body region. Data were collected on patient demographics and risk factors, including age, gender, American Society of Anesthesiologists (ASA) score, operating region, diabetes status, obesity, peripheral vascular disease, history of AFB infection, and HIV/AIDS. A microscopic exam was performed on all culture specimens. We evaluated whether the positive patients were placed on any medications, had revision surgery within one month of the positive culture, or had an amputation within one month of the positive result. Due to the small sample size in the positive AFB/fungal culture group, operative region was categorized as either hand or foot (including fingers or toes) versus all other operating regions.

All analyses were performed in R version 3.6.1 (R Foundation for Statistical Computing, Vienna, Austria). Surgical cases with versus without a positive AFB/fungal culture were compared using a Mann-Whitney U test for age and using Pearson’s Chi-Squared tests for all other variables. If any group size was less than five, a Fisher's Exact Test was used instead of a Pearson’s Chi-Squared test. For characteristics that significantly differed across study groups (peripheral vascular disease and operating region), logistic regression was used to estimate the odds of a positive culture result. First, unadjusted models were employed for each variable separately. Then, one adjusted model was used to explore the odds of positive culture result by both peripheral vascular disease and operating region.

## Results

There were 29,672 orthopaedic surgical cases performed between March 2013 and December 2019. Of these, 2.7% (813) had either an AFB or fungal culture ordered on the same day as their orthopaedic surgery. This included a microscopic evaluation. Of these 813 cases, 3.8% (N=31) had a positive result. Thirty positive results were from fungal cultures; one positive result was from an AFB culture.

The rate of positive culture results did not differ by age, sex, ASA score, diabetes status, obesity status, or HIV/AIDS (Table 1). Patients with peripheral vascular disease and patients with a hand or foot operating region had significantly higher rates of positive results (p=0.004 and p<0.001, respectively). Of the 55 patients with peripheral vascular disease, 13% (N=7) had a positive culture result. Patients without peripheral vascular disease had only a 3% positivity rate (24/758). Of the 293 patients with an operating region of hand or foot, 8% were positive (N=22), compared to only 2% of those with another operating region (9/520).

**Table 1:** Characteristics of patients with a positive vs. negative culture result ^1^ Of the 31 positive results, 30 were from fungal cultures and one was from an AFB culture. ^2^ P-values are from a Mann-Whitney U test for age, and from Pearson’s chi-squared tests for all other variables. If any group size was <5, Fisher's exact test was used instead of Pearson’s chi-squared test. ^3^ A total of 46 patients were missing ASA scores; 44 in the negative culture group and two in the positive culture group AFB: acid-fast bacilli; ASA: American Society of Anesthesiologists

	Culture Result		
	Negative fungal and AFB culture (782)	Positive fungal and AFB culture^1^ (31)	P-Value^2^
Average Age (SD)	58 (17)	57 (15)	0.46
Min, Max	2, 95	29, 90	
Sex			0.19
Male	62% (483)	48% (15)	
Female	38% (299)	52% (16)	
ASA Score^3^			0.13
1	6% (42)	10% (3)	
2	26% (193)	14% (4)	
3	57% (422)	55% (16)	
4	11% (81)	21% (6)	
5	0% (0)	0% (0)	
Operating Region			<0.001
Hand or Foot	34% (271)	71% (22)	
All Others	66% (511)	29% (9)	
Diabetes	26% (202)	35% (11)	0.32
Obesity	17% (131)	16% (5)	>0.99
Peripheral Vascular Disease	6% (48)	23% (7)	0.004
HIV or AIDS	0.5% (4)	0% (0)	>0.99

Of the patients with positive cultures, only one was prescribed an anti-fungal medication within a month of the positive result. Two patients had an amputation within one month of the positive result, and no reoperations were performed within one month of the results. 

In both unadjusted and adjusted models (Table 2), peripheral vascular disease and operative region were associated with higher odds of a positive culture result. When adjusting for the effect of the operating region, those with peripheral vascular disease had 3.5 times higher odds of a positive culture result (95%CI = 1.3-8.4). When adjusting for the effect of peripheral vascular disease, those with a hand or foot operating region had 4.2 times higher odds of a positive culture result (95%CI = 1.9-9.8) compared to those with another operating region.

**Table 2:** Odds of positive culture result by peripheral vascular disease and operating region ^1^ The adjusted model predicted the odds of a positive culture result by peripheral vascular disease and operating region

	Unadjusted Odds Ratio (95%CI)	Adjusted Odds Ratio^1^ (95%CI)
Peripheral Vascular Disease		
Yes	4.5 (1.7-10.4)	3.5 (1.3-8.4)
No	Reference	Reference
Operating Region		
Hand/Foot	4.6 (2.2-10.7)	4.2 (1.9-9.8)
Other	Reference	Reference

## Discussion

Our results support the low utility of routine AFB and fungal cultures for orthopaedic infections. Although these tests are often ordered, the rate of positive results is rare and should cause us to evaluate more carefully the reasons for ordering these labs.

Several studies have verified the low positivity rate for routine AFB and fungal cultures, which is also supported by our results [1-7]. Our study found a 3.8% positive culture result, which is higher than previous reports of 0-1.03%. This is likely because we included all orthopaedic-related infections. Many of the previous reports relate to prosthetic joint infections alone [1,2,7].

Although this study did not specifically evaluate the economic impact of routinely obtaining these labs, previous studies indicate significant cost savings by limiting the number of atypical cultures [7]. This would be dependent on the hospital system, geographic region, and varying lab costs associated with these cultures, which could be explored in future studies.

The rate of positive AFB in our study was negligible. The single patient who had a positive AFB culture result also had a known history of disseminated mycobacterium tuberculosis.

The clinical significance of a positive fungal or AFB culture result was minimal. Patient care did not seem to be affected by the positive fungal and AFB culture result. Only one patient was placed on anti-fungal medication after the positive result. Reoperation and type of procedure were not affected by the positive cultures. However, it was difficult to evaluate all factors for clinical significance based on this retrospective review. 

Our hypothesis was confirmed that PVD was associated with higher odds of a positive culture. We expected other medical comorbidities such as diabetes mellitus (DM) and obesity to be significant risk factors based on previous studies [1]. Although it is still possible these comorbid conditions play a role, we did not find them to be of statistical significance in this study.

In addition to PVD, an operating region of the hand or foot was associated with higher odds of a positive culture. Of patients in the hand and foot category, 8% were positive compared with only 2% in all other regions combined. This high positive rate in hand and foot operating regions could have also contributed to our study’s positive culture rate being higher than previously reported studies, which mainly assessed prosthetic joint infections. Our study included all orthopaedic cases including prosthetic joint infections. We are not aware of any literature to support why hand and foot regions might have a higher fungal infection rate. We speculate hands and feet are more readily accessible and exposed to infectious sources compared to other body regions. Onychomycosis is only seen in these areas and may play a role as well. The incidence of fungal nail disease in the United States is up to 14% [8]. Risk factors for onychomycosis included peripheral vascular disease and immunocompromised host [8]. This correlates with our study's results regarding risk factors for fungal infection. The Association of Perioperative Registered Nurses (AORN) has demonstrated higher bacterial counts with nails that have longer than a 2 mm distal free edge [9]. However, this did not specifically evaluate the presence of fungus or AFB. Artificial nails have also been shown to harbor more bacteria and fungus [10].

AFB and fungal cultures need to be ordered with careful consideration of the patient and potential risk factors for positive results. In the study by Wadey and colleagues, a new algorithm was created for obtaining cultures to limit their utilization of AFB and fungus [7]. In a similar way, our utilization of these tests should be tailored to patients with a higher risk of positive results such as patients with PVD and hand or foot operating regions. There is little to no utility in obtaining routine AFB cultures, except in patients with a history of tuberculosis.

There were several limitations to this study. First, the study was retrospective in nature and data was collected from electronic medical records. We may be facing missing or incomplete data from medical records. Second, there may be other risk factors worth consideration in our analysis, such as immunosuppressive medications, indwelling catheters, use of broad-spectrum antibiotics, parenteral hyperalimentation, organ transplantation, or other epidemiological risk factors [1-6]. We focused our data collection on factors that could be easily captured in our retrospective EMR collection of data. Third, we intentionally included all orthopaedic infections across all subspecialties to maximize our sample size. Future studies targeting specific subspecialties could be beneficial. It may also be useful to further examine foot and ankle or hand pathology in isolation, as this was found to be a risk factor for a positive result in the present study. Finally, because positive infection rates are low, the number of patients needed to find clinical and statistically significant differences across groups is high. We detected significant differences between groups when effect sizes were very large. Future studies with a larger sample size are warranted to explore smaller effect sizes.

## Conclusions

There is limited evidence establishing the utility of routine fungal and AFB cultures for orthopaedic infections, and most of the existing literature focuses on prosthetic joint infections. Our retrospective review of all orthopaedic surgical cases at one institution found a very low rate of positive routine AFB and fungal culture results. Intraoperative fungal cultures may not be indicated except in orthopaedic surgical cases for hand or foot infections and in patients with PVD. AFB cultures may not need to be obtained unless the patient has a history of tuberculosis.
